# Emerging Strategies to Protect the Skin from Ultraviolet Rays Using Plant-Derived Materials

**DOI:** 10.3390/antiox9070637

**Published:** 2020-07-18

**Authors:** Yong Chool Boo

**Affiliations:** Department of Molecular Medicine, School of Medicine, BK21 Plus KNU Biomedical Convergence Program, Cell and Matrix Research Institute, Kyungpook National University, Daegu 41944, Korea; ycboo@knu.ac.kr; Tel.: +82-53-420-4946

**Keywords:** sunlight, ultraviolet, oxidative damage, antioxidant, inflammation, melanin, photoprotection, photoaging, photocarcinogenesis, plant extract, cosmetics

## Abstract

Sunlight contains a significant amount of ultraviolet (UV) ray, which leads to various effects on homeostasis in the body. Defense strategies to protect from UV rays have been extensively studied, as sunburn, photoaging, and photocarcinogenesis are caused by excessive UV exposure. The primary lines of defense against UV damage are melanin and trans-urocanic acid, which are distributed in the stratum corneum. UV rays that pass beyond these lines of defense can lead to oxidative damage. However, cells detect changes due to UV rays as early as possible and initiate cell signaling processes to prevent the occurrence of damage and repair the already occurred damage. Cosmetic and dermatology experts recommend using a sunscreen product to prevent UV-induced damage. A variety of strategies using antioxidants and anti-inflammatory agents have also been developed to complement the skin’s defenses against UV rays. Researchers have examined the use of plant-derived materials to alleviate the occurrence of skin aging, diseases, and cancer caused by UV rays. Furthermore, studies are also underway to determine how to promote melanin production to protect from UV-induced skin damage. This review provides discussion of the damage that occurs in the skin due to UV light and describes potential defense strategies using plant-derived materials. This review aims to assist researchers in understanding the current research in this area and to potentially plan future studies.

## 1. Introduction

Solar energy is a major factor in the environment that interacts with life and has a positive or negative effect on the birth, growth, aging, and death of organisms [1]. Sunlight that passes through the atmosphere and reaches the earth’s surface mainly comprises visible light and some ultraviolet (UV) and infrared rays [2,3]. Of these, visible light and infrared rays are relatively safe for life and only have a harmful effect under special conditions, such as the presence of photosensitizers. However, high-levels of UV rays can lead to direct damage to living organisms.

In humans, melanin and trans-urocanic acid perform the primary defense functions in the skin by absorbing UV rays [4,5]. The body is equipped with various enzymatic and non-enzymatic measures to protect from the UV rays that pass through the primary lines of defense [6,7]. Damage is inevitable if UV rays are too intense or the defenses in the skin are not sufficient, leading to negative consequences, such as oxidative damage and disease. Therefore, enhancing the external defenses against UV rays, rather than relying only on the skin’s own defense capabilities, is essential to prevent UV-associated damage.

**Figure 1:**
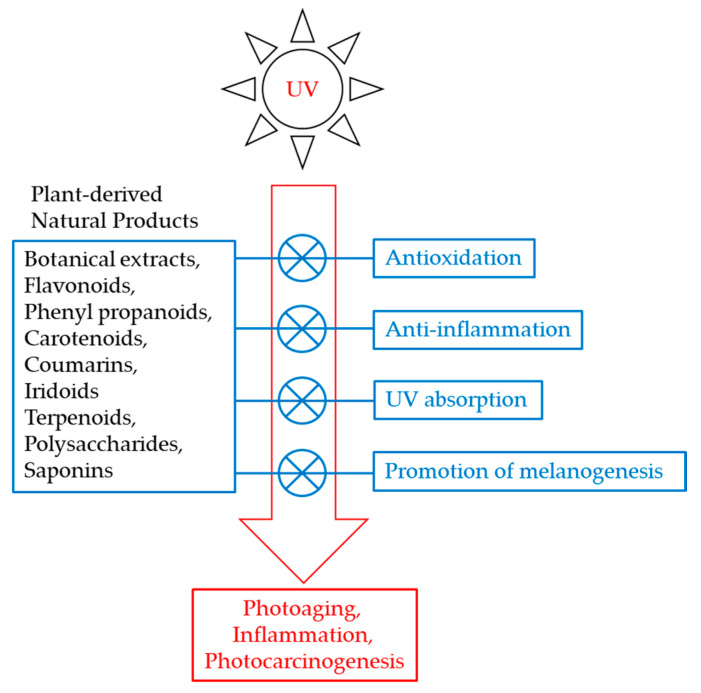
Scope of this review article. This review will cover emerging strategies to protect the skin from UV-induced toxic effects using plant-derived natural products which can act as UV absorbers, antioxidants, anti-inflammatory agents, and promoters of melanin synthesis.

Defense strategies to protect the skin against the harmful effects of UV rays have been widely studied [8]. In this review, we will introduce these studies, with a focus on protection against UV-induced toxicity using plant-derived natural products. These products are divided into several categories, including UV absorbers, antioxidants, anti-inflammatory agents, and promoters of melanin synthesis. Figure 1 shows the scope of this review. The aim of this review is to assist the development of novel research plans and industrial application strategies to reduce skin damage caused by UV rays.

## 2. UV-Induced Toxicity in the Skin

UV rays are categorized into UVA (315–400 nm), UVB (280–315 nm), and UVC (200–280 nm) rays, depending on the wavelength range (ISO 21348 Definitions of Solar Irradiance Spectral Categories). UV rays are a major cause of skin photoaging and photocarcinogenesis [9,10]. Overexposure to UV radiation, particularly the UVB component, causes erythema, edema, hyperplasia, hyperpigmentation, photoaging, immunosuppression, and skin cancer [11,12]. Overexposure of the skin to UV rays stimulates the production of reactive oxygen species (ROS); increases oxidative damage of biomolecules, such as lipids, nucleic acids, and proteins; and decreases endogenous antioxidants in the cutaneous tissues [13,14,15]. Approximately 9–14% of solar UVB rays reach the dermis in the skin and can induce inflammatory responses, such as erythema and edema [12]. Sunburn reactions are mediated by the tumor suppressor p53 [16,17]. The p53 arrests cell cycle, allowing cells to properly repair the damaged DNA or to remove the damaged cells, thereby reducing the risk of cancer development. Previous studies have demonstrated that exogenous antioxidants can prevent photocarcinogenesis [18].

Intrinsic aging of the human skin, also called natural or chronological aging, is dependent on time and genetics, whereas extrinsic skin aging is affected by environmental factors, such as solar radiation [19]. UV radiation is a major cause of skin photoaging, which is characterized by wrinkles, laxity, blister formation, roughness, and loss of skin tone [12,20,21]. Apart from intrinsic aging, which is currently inevitable, photoaging can be reduced by minimizing UV exposure and maintaining proper skin care [20]. The main issues that are targeted in the cosmetics field include wrinkles and unwanted pigmentation, which are associated with inflammation or oxidative stress due to overexposure to UV rays [12,20].

Skin photoaging involves alterations in the extracellular matrix composition of the dermis. UV rays induce and activate matrix metalloproteinases (MMPs), a group of zinc endopeptidases that degrade extracellular matrix macromolecules, including type I collagen [22,23]. MMPs secreted from both the dermal fibroblasts and epidermal keratinocytes participate in collagen metabolism in the skin [24,25]. MMPs play key roles in connective tissue remodeling in UV-exposed skin and cause wrinkles and other phenotypes in photo-aged skin [26,27]. 

Gene expression of MMPs, such as MMP-1, -2, -3, and -9, is upregulated in UV-exposed human dermal fibroblasts [26,27]. The cell signaling pathways involve UV-induced activation of cytokine receptors and the subsequent activation of mitogen-activated protein kinases (MAPK), such as extracellular signal-regulated kinase (ERK), c-Jun-N-terminal kinase (JNK), and p38 kinase [28,29,30]. The promoters of the MMP-1 and MMP-3 genes can be transactivated by activator protein-1 (AP-1) complexes [29,31]. Although the initial events of this signal cascade are not fully understood, evidence suggests that UV-damaged DNA acts as a trigger that initiates this process [32]. 

Keratinocytes account for 95% of the mass of cells in the human epidermis and play an important role in maintaining skin homeostasis, through both their autocrine and paracrine effects [33]. Under normal conditions, the constitutive production of cytokines and other soluble factors in human keratinocytes is low; however, various stimuli, such as UV rays and endotoxins, can trigger the expression of pro-inflammatory cytokines [34]. Certain cytokines or cell components that are secreted from UVB-irradiated epidermal keratinocytes can regulate gene expression of dermal fibroblasts through paracrine effects. For example, interleukin (IL)-1β, tumor necrosis factor (TNF)-α, and stratifin are released from UV-exposed keratinocytes and stimulate MMP-1 expression in fibroblasts [35,36,37].

Apoptotic cell death is involved in skin photoaging [38], and typically involves changes in the expression of the pro-apoptotic (Bax, Bak, and Bid) and anti-apoptotic (Bcl-2 and Bcl-x) members of the Bcl-2 protein family [39]. UV-induced apoptosis is mediated by caspases in keratinocytes [40]. UV rays induce apoptosis of keratinocytes via intrinsic pathways, involving direct DNA damage; extrinsic pathways, involving activated cell membrane death receptors; and other ROS-mediated pathways [38,40]. Apoptosis can be detected using various markers, including DNA laddering, changes in the expression of pro-apoptotic (Bax, Bak, and Bid) and anti-apoptotic (Bcl-2 and Bcl-x) members of the Bcl-2 protein family, and activation of caspases [41,42,43].

## 3. Melanin as an Endogenous UV Filter

Melanin is a polymeric dark pigment produced by melanocytes [44]. Pheomelanin and eumelanin are the major forms of melanin that are found in the skin, hair, iris of eyes, and the stria vascularis of the inner ear, whereas neuromelanin is found in the brain. In human skin, epidermal melanocytes are present at the junction of the dermis and epidermis [44]. The number of melanocytes per unit area of skin does not vary greatly among individuals; however, melanocytes from individuals with different skin colors have different activities that lead to more or less production of pheomelanin or eumelanin [12,45]. 

There is a close relationship between the melanogenic activity and human skin color [46,47]. The vertical and horizontal distribution of melanin in the skin can also change the appearance of the skin color [48]. Skin color also appears to be associated with genetic background, e.g., mutations in the *SLC24A5* and *SLC45A2*, which encode solute carrier proteins [49,50]. Single nucleotide polymorphisms in these genes alter the activity of the potassium-dependent sodium–calcium exchanger and the biogenesis of melanosomes [51,52]. Other intrinsic and extrinsic factors contribute to skin color by regulating the expression of melanin-related genes [53]. 

Abnormal melanin metabolism can lead to skin pigment disorders, which are categorized into either hyperpigmentation or hypopigmentation [53,54]. Hyperpigmentation occurs when melanin excessively accumulates owing to various internal and external stimulatory factors [55,56]. Meanwhile, hypopigmentation occurs when melanin production is reduced by genetic or epigenetic factors, as observed in albinism or vitiligo [57,58]. 

Melanin plays an important role in the regulation of epidermal homeostasis which is associated with the behavior of melanocytes [45,59,60]. Melanin absorbs UV radiation and dissipates energy in the form of heat, providing protection against UV radiation in the skin [10]. In our in vitro study, small interfering RNA (siRNA)-mediated knockdown of tyrosinase (TYR) resulted in decreased melanin content and viability of melanocytes exposed to UV rays [61]. The incidence of malignant melanoma is known to be significantly lower in dark-skinned people than in fair-skinned people [62]. 

## 4. Trans-Urocanic Acid and Sunscreen Products

trans-Urocanic acid is a major acid-soluble UV-absorbing compound in the stratum corneum [63]. The photon energy absorbed by trans-urocanic acid is dissipated in the form of heat in a reversible isomerization reaction to its cis isomer [64,65,66]. Histidase-deficient mice that cannot produce urocanic acid are more prone to UV-induced DNA damage and apoptotic cell death than the wild type littermates, supporting an essential role of urocanic acid in UV protection [67]. However, there is a controversy surrounding whether topically applied urocanic acid has a beneficial or detrimental effect when the skin is exposed to UV radiation [68]. Urocanic acid has been shown to mediate an immunosuppressive effect against UV rays [69] and increase photo-carcinogenic risk in hairless mice [70]. 

Sunscreen products are widely used for the maintenance of skin health and beauty [71]. Cosmetic and dermatology experts recommend using a sunscreen product to assist the skin’s own defense against UV rays. Although current evidence suggests that both inorganic and organic agents in sunscreen products are safe enough for daily use on the skin, there is an increasing concern regarding the penetration of sunscreen agents into the skin and the potential harmful side effects of these products [72,73]. Therefore, there is a need for the development of safer and more effective strategies for UV protection. 

## 5. UV Protection by Botanical Extracts 

Numerous plant extracts or constituents have previously been demonstrated to attenuate inflammatory responses due to UV exposure in cells, animals, and humans [74,75]. Selected studies have investigated plant extracts and the key findings of these studies are listed in Table 1. 

In our study, botanical extracts derived from *Sasa quelpaertensis*, *Althaea rosea*, and *Dryopteris crassi rhizoma* attenuated cytotoxicity and melanin synthesis in cultured human epidermal melanocytes exposed to UVB rays [84]. When these plant extracts were topically applied to the ears of C57BL/6 mice or the dorsal skin of the SKH-1 hairless mouse before and after exposure to UVB rays, they prevented an increase in ear thickness or dorsal skin redness, suggesting that they possess anti-inflammatory activity that reduces edema and erythema [84]. *Sasa quelpaertensis* extract, which contains *p*-coumaric acid as one of its main constituents, had the most potent activity [76,84]. 

*Bambusae caulis in Taeniam* has been used as a health food additive and a traditional medicine for the treatment of atherosclerosis, hyperlipidemia, hypertension, and fatigue, among other conditions [85,86,87]. Its bioactivity is believed to be at least partly related to potent antioxidant properties [24,88]. *Bambusae caulis in Taeniam* extract, which also contains *p*-coumaric acid, enhanced the viability of UVB-exposed HaCaT human keratinocytes and attenuated apoptotic events, including the cleavage of procaspase 3 to its active form and an increase in the Bax to Bcl-2 ratio [42]. It also exhibited antioxidant activity by decreasing the generation of ROS and reducing lipid peroxidation in cells exposed to UVB [42]. Additionally, it reduced the expression of MMP1 and phosphorylation of JNK after stimulation with UVB [42]. 

We have also compared the protective effects of a number of yellow plant extracts, such as *Gardenia jasminoides*, *Phellodendron amurense*, and *Rheum rhabarbarum*, in HaCaT keratinocytes exposed to UVB rays [41]. Of the plant extracts tested, *Gardenia jasminoides* extract had the lowest cytotoxicity and enhanced the viability of UVB-exposed cells in a dose-dependent manner. The extract also attenuated lipid peroxidation, the gene expression of IL-1β, TNF-α and MMP1, and UVB-induced apoptosis, supporting its antioxidative, anti-inflammatory, and anti-apoptotic effects. Many of these properties that *Gardenia jasminoides* extract exhibits against UV treatment are attributed to crocin, a water-soluble carotenoid derivative [89]. The pharmacological effects of crocetin and crocin have been widely investigated [90,91]. 

*Scutellaria radix*, which is the root of *Scutellaria baicalensis* Georgi, has been used in traditional medicine in Asia to treat inflammatory and allergic diseases because it contains various flavonoids, such as bailcalein and baicalin (baicalein-7-*O*-glucuronide) [92]. *Scutellaria radix* extract and its constituents have been shown to exhibit antioxidant and anti-inflammatory effects in various experimental models [93,94,95]. *Scutellaria radix* extract also showed UV-protective effects [96,97,98]. In our study, the extract of *Scutellaria radix* showed high UV absorptivity and free radical scavenging activity, and attenuated UV-induced cell death of HaCaT keratinocytes [82]. The inclusion of the *Scutellaria radix* extract in sunscreen cream significantly enhanced the sun protection factor (SPF), as determined in human subjects [82]. 

Propolis is a mixture of pollen, resin, bee wax, and salivary gland secretions produced by honeybees and contains various phenolic compounds [99]. Previous studies have shown that propolis and its constituents exhibited antioxidant effects via mitigating oxidative modifications of biomolecules [77,100]. Propolis extract has also been shown to have immunomodulatory and anti-inflammatory effects under various pathological conditions [77,101,102]. In a recent study, propolis extract significantly lowered the total protein carbonyl content, a marker of protein oxidation, in HaCaT cells exposed to UVB rays [83]. It also attenuated oxidative photodamage due to UVB exposure in a model of reconstituted skin tissue [83].

An extract of *Portulaca oleracea*, commonly called Purslane [103], has been shown to reduce apoptotic cell death of human fibroblasts and keratinocytes after UVB irradiation, as mitochondrial membrane depolarization was detected by JC-1 staining, phosphatidylserine exposure was detected by annexin V-fluorescein isothiocyanate (FITC) staining, and apoptotic DNA fragmentation was detected using the terminal deoxynucleotidyl transferase dUTP nick end labeling (TUNEL) assay and electrophoretic DNA ladder assay [80]. Other extracts derived from broccoli sprouts, blackberries, citrus, and rosemary have also been shown to have UV-protective effects in keratinocytes, mice, and humans [78,79,81]. 

## 6. Plant-Derived Antioxidants That Protect Melanocytes

Melanocytes localized in the stratum basale of the epidermis can also be exposed to UV rays that can increase ROS generation in the cell and deplete the endogenous pool of antioxidants [104]. UV-induced oxidative stress of melanocytes is related to the occurrence of vitiligo and melanoma [105,106]. Mimicking these conditions by exposing melanocytes to hydrogen peroxide and using various defense strategies with antioxidants have been important research topics in the field of dermatological sciences. 

As listed in Table 2, numerous studies have reported that a variety of plant-derived compounds, including flavonoids, such as quercetin, apigenin, (−)-epigallocatechin-3-gallate, hyperoside, afzelin, and baicalein; iridoids, such as geniposide; and terpenoids, such as bilobalide, alleviated oxidative stress and reduced apoptosis in melanocytes exposed to hydrogen peroxide. This experimental evidence supports the working hypothesis that certain plant-derived antioxidants may alleviate the oxidative stress of melanocytes in human skin exposed to UV rays.

## 7. UV Absorption by Phenyl Propanoids 

**Figure 2:**
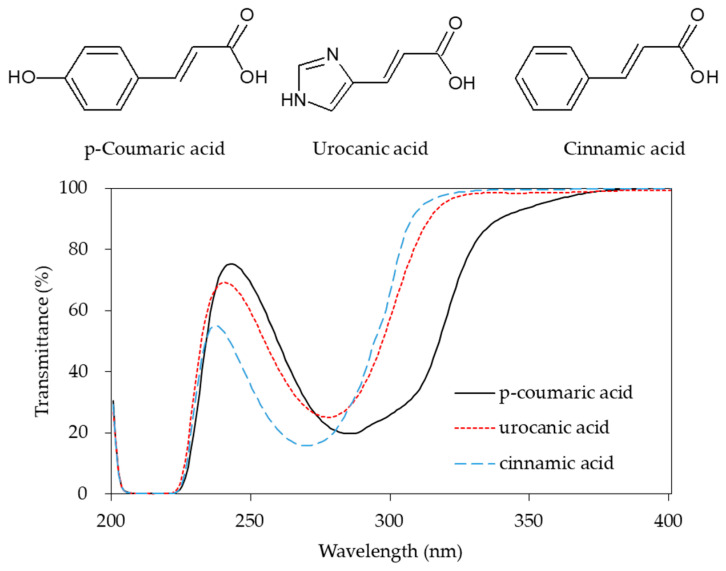
Chemical structures and ultraviolet (UV) transmittance spectra of *p*-coumaric acid, urocanic acid, and cinnamic acid. The compounds were dissolved in phosphate buffered saline at 30 μM.

Urocanic acid is synthesized by L-histidine ammonia lyase (also called histidase) from histidine [116]. Botanical compounds, *p*-coumaric acid, and cinnamic acid are synthesized by L-tyrosine ammonia lyase from L-tyrosine, and by L-phenylalanine ammonia lyase from L-phenylalanine, respectively [117,118]. The chemical structures and UV transmittance spectra of *p*-coumaric acid, urocanic acid, and cinnamic acid are presented in Figure 2. These compounds can act as effective UV filters, with *p*-coumaric acid having the broadest spectrum. 

*p*-Coumaric acid is a common secondary metabolite in plants that has been shown to have antioxidant activity in a variety of oxidative stress models, including cell [119] and animal [120,121] models. *p*-Coumaric acid attenuated UVB toxicity in human epidermal melanocytes [122] and HaCaT human keratinocytes [123]. *p*-Coumaric acid also reduced erythema induction in hairless mice and human skin exposed to UV [123,124,125]. Certain protein factors released from UV-irradiated keratinocytes induced MMP-1 expression in dermal fibroblasts via paracrine effects [36,126]. Keratinocyte-releasable stratifin was shown to induce MMP-1 expression in target fibroblasts [127,128]. In our study, *p*-coumaric acid reduced the expression and secretion of stratifin and indirectly attenuated MMP-1 expression in fibroblasts in medium transfer experiments [43]. 

Cinnamic acid attenuated UVA-induced expression of MMP-1 and -3 and the degradation of type I procollagen through inhibition of AP-1 and induction of nuclear factor erythroid 2-related factor 2 (Nrf2)-mediated antioxidant gene expression in human dermal fibroblasts [129]. Caffeic acid and ferulic acid showed anti-inflammatory and anticarcinogenic effects in mice exposed to UV [130,131,132,133,134]. The incorporation of ferulic acid in a sunscreen product as an anti-inflammatory additive increased SPF and UVA-protection factor (UVA-PF), as demonstrated in human skin [135]. Selected studies on the protective effects of cinnamic acid, *p*-coumaric acid, caffeic acid, and ferulic acid against UV rays are listed in Table 3.

## 8. Anti-inflammatory and Anticarcinogenic Effects of Quercetin

Flavonoids are a group of phenolic compounds derived from plants [136]. Various flavonoids have diverse bioactivities depending on their chemical structure, and the uptake of certain flavonoids is believed to have health benefits [137,138]. In this section, studies on the anti-inflammatory and anticarcinogenic effects of quercetin, a representative flavonoid, will be discussed.

In 1997, Steerenberg et al. reported that the oral administration of quercetin had no effect on the onset or growth of non-melanoma skin tumors in SKH hairless mice exposed to sub-erythemal doses of UVB for 17 weeks, although quercetin treatment restored the skin-associated contact hypersensitivity to picryl chloride [139,140]. Subsequent studies by Erden Inal et al. showed that intraperitoneal administration of quercetin reduced oxidative stress in Sprague–Dawley rats exposed to UVA for 9 days [141]. Casagrande et al. reported that the topical application of quercetin, formulated in emulsions, attenuated UVB-induced skin damage in hairless mice (HRS/J) [142]. 

Quercetin has also been shown to inhibit UV-induced lipid peroxidation in liposomes in vitro, primarily by scavenging UV-generated radical species, although it can also absorb UV radiation [143]. Quercetin decreased UV-induced nuclear factor (NF)-κB activation in HaCaT keratinocytes, thereby suppressing gene expression of inflammatory cytokines, such IL-1β, IL-6, IL-8, and TNF-α [144]. Quercetin has also been shown to lower the levels of ROS generation in HaCaT keratinocytes exposed to UVB and prevent the loss of cell membrane fluidity, mitochondrial membrane depolarization, outflow of cytochrome C, and apoptosis [145]. 

Quercetin-loaded nanoparticles prepared using poly(D,L-lactide-co-glycolide) (PLGA) and tocopheryl polyethylene glycol 1000 succinate (TPGS), suppressed UVB-induced NF-kB activation and cyclooxygenase (COX) 2 expression in HaCaT keratinocytes [146]. The quercetin-loaded PLGA-TPGS nanoparticles exhibited enhanced skin permeation and protective effects against UVB-induced damage in the skin of mice [146]. Quercetin-loaded chitosan was shown to permeate through the cell membrane and to inhibit the NF-kB/COX-2 signaling pathway in HaCaT keratinocytes, without affecting cell viability [147]. It also enhanced the percutaneous penetration of quercetin and modulated the NF-kB/COX-2 signaling pathway, ameliorating skin edema in C57BL/6 mice exposed to UVB irradiation [147].

Based on the findings of these studies, it has been suggested that quercetin has the potential to be used in dermatological or cosmetological approaches to attenuate oxidative stress and inflammation of the skin due to exposure, although its activity may not be sufficient to exert anticarcinogenic effects.

## 9. Synthesis of Melanin

Melanin is synthesized through a series of oxidative reactions inside specialized organelles called melanosomes [148,149]. Proopiomelanocortin-derived peptide hormones, such as α-melanocyte stimulating hormone (MSH), β-MSH, and adrenocorticotrophic hormone, stimulate skin pigmentation in response to UV and/or inflammatory stimuli [53,150]. 

The binding of an agonist to the melanocortin 1 receptor (MC1R), a G protein-coupled receptor, initiates a series of signaling events. The activation of adenylate cyclase produces cAMP, which in turn activates protein kinase A (PKA), which phosphorylates and activates cAMP-responsive element-binding protein (CREB); the CREB transcription factor then induces microphthalmia-associated transcription factor (MITF) gene expression and activation [151]. In addition to the α-MSH/MC1R pathway, the stem cell factor (SCF)/tyrosine kinase receptor c-Kit/MAPK pathway and the Wnt/Frizzled/glycogen synthase kinase (GSK) 3β/β-catenin pathway can activate MITF [152,153]. Other intracellular signaling pathways, such as phospholipase C (PLC)/diacyl glycerol (DAG)/protein kinase C (PKC) β cascade and nitric oxide (NO)/cGMP/protein kinase G (PKG) cascade, are also involved in the regulation of melanogenesis [154,155].

MITF controls the biogenesis of melanosomes as well as the gene expression of TYR, tyrosinase-related protein 1 (TYRP1), and dopachrome tautomerase (DCT) in melanocytic cells [44,156]. TYR catalyzes the initial steps of melanin synthesis by converting L-tyrosine and L-dihydroxyphenylalanine (DOPA) to L-dopaquinone [157]. These reactions are followed by subsequent reactions, which may involve thiol conjugations, leading to the synthesis of reddish-yellow pheomelanin or brownish black eumelanin [158]. 

Mature melanosomes that contain melanin pigments are delivered from a single melanocyte to several tens of epidermal keratinocytes in close proximity, spreading melanin pigments throughout the epidermis [159]. 

## 10. Use of MC1R Agonists to Stimulate Melanin Synthesis

Previous studies have investigated a strategy for melanoma prevention through the enhancement of eumelanin synthesis using α-MSH analogs that function as MC1R agonists [160]. [Nle4-D-Phe7]-α-MSH is the first synthetic analog of α-MSH [161]. It is a linear 13 amino acid peptide and its amino sequence, Ac-Ser-Tyr-Ser-Nle-Glu-His-D-Phe-Arg-Trp-Gly-Lys Pro-Val-NH_2_, is similar to that of α-MSH. The methionine at the 4th position and L-phenylalanine at the 7th position of α-MSH are replaced by norleucine and D-phenylalanine, respectively, to enhance resistance to enzymatic degradation. [Nle4-D-Phe7]-α-MSH alone or in combination with UV radiation induces human skin tanning [162]. Phase II trials found that treatment with [Nle4-D-Phe7]-α-significantly increased melanin density and tolerance to artificial light [163].

Abdel-Malek et al. showed that tetrapeptide analogs of α-MSH, such as Ac-His-D-Phe-Arg-Trp-NH_2_, n-Pentadecanoyl-His-D-Phe-Arg-Trp-NH_2_, and 4-Phenylbutyryl-His-D-Phe-Arg-Trp-NH_2_, enhanced melanin synthesis and promoted human melanocyte survival under conditions of UV irradiation [164]. Jackson et al. identified tetrapeptide analogs of α-MSH that function as highly selective MC1R agonists [165]. They showed that the pentapeptides Bz-Gly-His-D-Phe-AA_B_-AA_A_-NR_1_R_2_ exhibited potency similar to that of [Nle4-D-Phe7]-α-MSH. In an ex vivo experiment on human skin tissue culture, Bz-Gly-His-D-Phe-D-Arg-D-Trp-N(CH_2_CH_2_CH_3_)_2_ induced the protein expression of MITF, TYR, and TYRP-1 and enhanced the activation of Nrf2 after UVA-irradiation. Further studies are needed to determine the in vivo efficacy of these melanogenic peptides. 

## 11. Plant-Derived Materials that Stimulate Melanin Synthesis

The induction of melanin synthesis is an attractive strategy to alleviate UV-induced damage in the skin [166]. Table 4 shows selected studies that investigated botanical extracts and their constituents that were found to promote melanin synthesis in cells. 

Forskolin is a diterpene compound isolated from the roots of *Coleusforskohlii forskohlii*, and it is a potent activator of adenylate cyclase [167]. Increased melanin levels, as a result of forskolin treatment, prevented the incidence of skin cancer in mice exposed to UV irradiation [168]. The promotion of melanin synthesis could alleviate UV-induced damage that causes skin photoaging and photocarcinogenesis. 

Flavonoids, such as pratol [169], apigenin-7-butylene glucoside [170], liquiritin, and liquiritigenin [171], and coumarins, such as umbelliferone [172], promoted melanin synthesis in B16 F10 mouse melanoma cells. Some of these compounds activated the p38, JNK, and PKA signaling pathways. However, it is uncertain whether the rest of the compounds have similar mechanisms of action. 

*Gynostemma pentaphyllum* saponins induced melanogenesis and activated the cAMP/PKA and Wnt/β-catenin signaling pathways [173], and *Cistanche deserticola* polysaccharides induced melanogenesis via activation of the MAPK signaling pathway and upregulation of MITF [174]. The extracts of *Melia azedarach* [175] and *Argania Spinosa* [174] induced melanin synthesis via activation of the cAMP signaling pathways in independent studies. 

To date, most of the studies that have been conducted have shown that certain botanical products promote melanin production at the cellular level. Further studies are needed to examine whether increased melanin levels improve the resistance of cells to oxidative damage due to UV exposure. The identification of active constituents of these plant extracts and the exact mechanisms of action remains to be elucidated.

## 12. Plant-Derived Materials that Attenuate Extrinsic Skin Aging

A number of plant extracts and constituents have been shown to suppress the gene expression of MMPs in dermal fibroblasts and epidermal keratinocytes exposed to environmental factors including UV radiation and airborne particulate matters (PM), indicating their potential skin antiaging effects. As mentioned above, *Bambusae caulis in Taeniam* extract and *p*-coumaric acid, and *Gardenia jasminoides* extract attenuated the expression of MMP-1 in UVB-exposed HaCaT human keratinocytes [41,42]. Additionally, *Quercus glauca* extract and rutin (quercetin-3-*O*-rutinoside) inhibited the UVB-induced expression of MMP-1 in human dermal fibroblasts [177]. Geniposide was shown to attenuate UV-B-induced photooxidative stress and MMP-2 expression in human dermal fibroblasts [178].

In human epidermal keratinocytes exposed to PM, (−)-epigallocatechin gallate and punicalagin lowered the mRNA expression of MMP-1 [179]. (−)-Epigallocatechin gallate also decreased the expression of MMP-1, -2, -8, -9, and -13 in human dermal fibroblasts exposed to PM [180]. *Camellia japonica* flower extract inhibited urban dust-induced MMP-1 expression in cultured human dermal fibroblasts and in human skin explants [181]. Therefore, certain plant-derived materials can attenuate the extrinsic skin ageing process by suppressing the expression of MMPs involved in collagen degradation. For additional studies on the antiaging effects of plant-derived compounds, please see other review articles [182,183,184]. 

## 13. Conclusions

The hypothesis that plant-derived materials with UV-absorbing, antioxidant, anti-inflammatory, and melanin synthesis-promoting properties will alleviate UV-induced toxicity is supported by a variety of experimental evidence from in vitro and in vivo studies. Various plant-derived compounds, such as phenyl propanoids, flavonoids, and carotenoids, can absorb UV rays and release energy in the form of heat. These compounds can also act as antioxidants by directly scavenging various types of free radicals or by enhancing the intrinsic antioxidant capacity through the Nrf2-dependent pathway. Certain plant-derived compounds, such as quercetin, can also suppress the amplification of UV toxicity by inhibiting target enzymes involved in inflammation. 

Melanocytes can be damaged by UV toxicity; however, these cells have the unique function of synthesizing melanin, which can assist in the protection of all types of skin cells, including keratinocytes, fibroblasts and melanocytes themselves. If melanocytes die or their ability to synthesize melanin decreases, the skin’s UV defense capability weakens. In other words, restoring the survival of melanocytes exposed to oxidative stress or promoting melanin synthesis will improve the overall UV tolerance capacity of the skin. Various flavonoids, iridoids, and terpenoids have been shown to alleviate oxidative stress and apoptosis of melanocytes exposed to hydrogen peroxide. Furthermore, various natural products, such as flavonoids, coumarins, polysaccharides, and saponins, have been shown to promote melanin synthesis in melanocytes. 

Upon UV exposure, melanin synthesis in melanocytes acts as a defensive measure for all skin cells. After enough melanin is produced, the melanin acts as a filter or shield against UV light. However, during the period of melanin synthesis, when the melanin levels are not high enough for protection, the skin still has a high risk of UV-induced damage. This means that UV-assisted tanning can harm the skin and that “sunless tanning” is a better choice [160,168]. Natural products that can preserve melanocyte viability under conditions of oxidative stress and can induce melanin synthesis in the absence of UV radiation will provide a preemptive defense against UV exposure. 

The regulation of melanin metabolism in the skin is important not only for skin health but also for cosmetic purposes. A substance that rescues the viability of melanocytes and stimulates melanin synthesis will be also useful in the prevention and treatment of hypopigmentation diseases, such as vitiligo [185]. Conversely, these substances may not be preferable for those who want to have a clean and light skin tone, as the promotion of melanin synthesis can lead to hyperpigmentation. 

Plant-derived ingredients can be misunderstood to be safe because they are natural. However, certain plant-derived compounds can act as prooxidants rather than antioxidants depending on the situation [186]. In addition, they can induce cytotoxicity, cell death, inflammation, metabolic disturbance, and carcinogenesis in certain circumstances [187]. Therefore, every plant-derived component must be sufficiently examined from a toxicological aspect before use.

**Figure 3:**
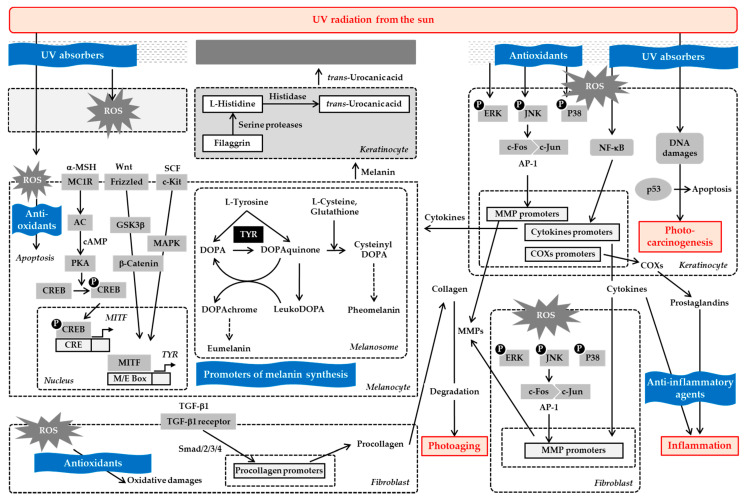
Emerging strategies using plant-derived materials to protect the skin from ultraviolet (UV)-induced damage. Overexposure of the skin to UV radiation induces production of reactive oxygen species (ROS), gene expression of matrx metalloproteinases (MMPs), cytokines, and cyclooxygenases (COXs), p53 activation, and oxidative damages in DNA and other biomolecules. These can lead to photoaging, inflammation, apoptosis, and/or photocarcinogenesis. Melanin and trans-urocanic acid provide UV-defensive measures. Research has shown that various plant-derived materials, such as botanical extracts, flavonoids, phenylpropanoids, carotenoids, coumarins, iridoids, terpenoids, polysaccharides, and saponins, can help mitigate UV-induced toxicity. Plant-derived materials can act as UV absorbers, antioxidants, anti-inflammatory agents, and/or promoters of melanin synthesis. Future clinical studies are needed to determine whether these UV defense strategies using plant-derived materials can reduce the incidence or progression of photoaging, inflammation, and photocarcinogenesis in humans.

This review introduced emerging strategies for reducing UV toxicity using plant-derived materials (Figure 3). There are various substances that have been demonstrated to prevent UV-induced oxidative damage in epidermal keratinocytes or dermal fibroblasts, attenuate the death of epidermal melanocytes under oxidative stress conditions, and promote “sunless” melanin synthesis in melanocytes. Although future studies are required to verify the in vivo and clinical efficacy of these substances, emerging strategies using these plant-derived materials are expected to open new possibilities for the prevention of skin photoaging and photocarcinogenesis.

## Figures and Tables

**Table 1:** Protective effects of plant-derived extracts against ultraviolet (UV) radiation-induced toxicity.

Models	Materials	Key Findings	Literature
C57BL/6 mice, SKH-1 hairless mice	*Sasa quelpaertensis*	Topically applied plant extracts reduced edema and erythema in mice exposed to UV light.	[76]
SKH:hr-1 hairless albino mice	Propolis	The extract reduced cutaneous inflammation, immunosuppression, and lipid peroxidation induced by UV exposure.	[77]
SKH-1 hairless mice	Broccoli sprout	Dietary glucoraphanin-rich broccoli sprout extracts protected against UV-induced skin carcinogenesis.	[78]
Primary keratinocytes	Blackberry	Anthocyanin-rich fractions of blackberry extracts reduced UV-induced free radicals and oxidative damage in cells.	[79]
HaCaT human keratinocytes	*Gardenia jasminoides*	The extract displayed antioxidant, anti-inflammatory, and anti-apoptotic effects.	[41]
Human epidermal keratinocytes, Human dermal fibroblasts	*Portulaca oleracea*	The extracts protected human keratinocytes and fibroblasts from UV-induced apoptosis.	[80]
HaCaT human keratinocytes, Human volunteers	Citrus and Rosemary	The extracts protected UV-induced damage in a skin cell model and in human volunteers.	[81]
HaCaT human keratinocytes	*Bambusae caulis in Taeniam*	The extract enhanced the viabilities of UVB-exposed cells and reduced the number of apoptotic events.	[42]
HaCaT human keratinocytes, Humans volunteers	*Scutellaria radix*	The extract enhanced the sun protection factor (SPF) of a sunscreen product, as determined in human subjects.	[82]
HaCaT human keratinocytes, Reconstituted human skin tissue	Propolis	The extract inhibited UV-induced photodamage.	[83]

**Table 2:** Cytoprotective effects of plant-derived antioxidants in melanocytes.

Materials	Models	Key Findings	Literature
Quercetin 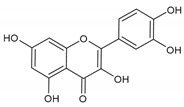	Mel-Ab melanocytes	Quercetin reduced H_2_O_2_-induced cell death.	[107]
	Normal human epidermal melanocytes	Quercetin attenuated ER dilation and H_2_O_2_-induced apoptosis.	[108]
Apigenin 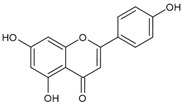	Normal human epidermal melanocytes	Apigenin attenuated dopamine-induced apoptosis.	[109]
Hyperoside 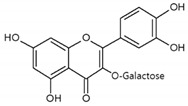	Normal human epidermal melanocytes	Hyperoside (quercetin-3-*O*-galactoside) decreased apoptosis of H_2_O_2_-injured melanocytes.	[110]
(−)-Epigallocatechin-3-gallate 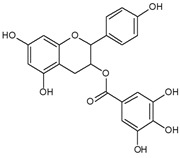	Normal human epidermal melanocytes	(−)-Epigallocatechin-3-gallate decreased apoptosis in H_2_O_2_-injured melanocytes.	[111]
Afzelin 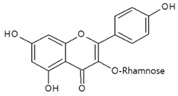	Normal human epidermal melanocytes	Afzelin (kaempferol-3-*O*-rhamnoside) inhibited H_2_O_2_-mediated cell death.	[112]
Baicalein 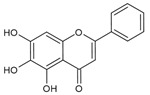	Human vitiligo melanocytes	Baicalein inhibited H_2_O_2_-induced cytotoxicity and apoptosis.	[113]
Geniposide 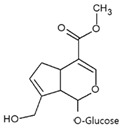	Normal human epidermal melanocytes	Geniposide (genipin-1-*O*-glucoside) decreased the apoptosis rate of H_2_O_2_-treated cells.	[114]
Bilobalide 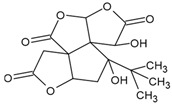	Normal human epidermal melanocytes	Bilobalide attenuated H_2_O_2_-induced apoptosis and ER stress.	[115]

**Table 3:** Protective effects of phenyl propanoids against UV-induced toxicity.

Compounds	Models	Key Findings	Literature
Cinnamic acid 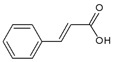	Human dermal fibroblasts	Cinnamic acid attenuated UVA-induced metalloproteinase expression through inhibition of AP-1 and activation of Nrf2.	[129]
*p*-Coumaric acid 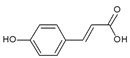	Human epidermal melanocytes	*p*-Coumaric acid inhibited melanin synthesis and attenuated UVB toxicity in melanocytes.	[122]
	Human epidermal melanocytes, Mice	*p*-Coumaric acid reduced erythema and pigmentation in the skin of mice exposed to UV rays.	[124]
	Humans	*p*-Coumaric acid reduced erythema and pigmentation in human skin exposed to UV rays.	[125]
	HaCaT human keratinocytes, Mice	*p*-Coumaric acid attenuated UVB toxicity in keratinocytes and reduced erythema and edema in mice skin exposed to UV rays.	[123]
Caffeic acid 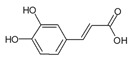	Mice	Caffeic acid suppressed UVB radiation-induced expression of interleukin-10 and activation of MAPKs involved in contact hypersensitivity.	[130]
	Mice	Caffeic acid targeted ERK1/2 to attenuate solar UV-induced skin carcinogenesis.	[131]
	Mice	Caffeic acid prevented UVB-induced photocarcinogenesis through regulation of PTEN signaling.	[132]
Ferulic acid 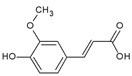	Mice	Ferulic acid suppressed UVB-induced MMP-2 and -9 expression in mouse skin.	[133]
	Mice	Intraperitoneal and topical administration of ferulic acid reduced the incidence of UVB-induced tumors.	[134]
	Humans	Ferulic acid incorporated in a sunscreen product increased SPF and UVA-PF in human skin.	[135]

**Table 4:** Induction of melanogenesis by plant-derived materials.

Models	Materials	Key Findings	Literature
C57BL/6 mice	Forskolin 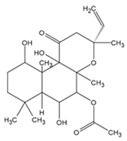	Forskolin-induced pigmentation was protective against UV-induced cutaneous DNA damage and tumorigenesis.	[168]
B16F10 mouse melanoma cells	Pratol 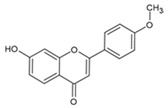	The compound induced melanogenesis via upregulation of phospho-p38 and phospho-JNK.	[169]
	Umbelliferone 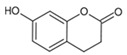	The compound stimulated melanogenesis and increased glutathione levels in cells.	[172]
	Apigenin-7-butylene glucoside 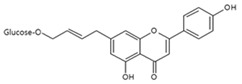	The compound induced melanogenesis by increasing tyrosinase activity in cells.	[170]
	*Gynostemma pentaphyllum*	Its saponins induced melanogenesis and activated the cAMP/PKA and Wnt/β-catenin signaling pathways.	[173]
	*Argania Spinosa*	Its fruit shell extract induced melanogenesis via activation of the cAMP signaling pathway.	[176]
B16F10 mouse melanoma cells, Human melanoma cell lines (HMVII)	Liquiritin and liquiritigenin 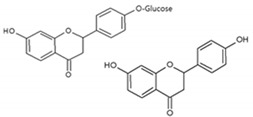	The compounds induced melanogenesis via enhancement of the p38 and PKA signaling pathways.	[171]
B16F10 mouse melanoma cells, Human epidermal melanocytes	*Melia azedarach*	Its ethanolic extract induced melanogenesis through the cAMP/PKA/CREB signaling pathway.	[175]
	*Cistanche deserticola*	Its polysaccharides induced melanogenesis via activation of MAPK signaling pathway and upregulation of MITF.	[174]

## Abbreviations

AP-1	activator protein-1
COX	cyclooxygenase
CREB	cAMP-responsive element-binding protein
DAG	diacyl glycerol
DOPA	dihydroxyphenylalanine
DCT	dopachrome tautomerase
ERK	extracellular signal-regulated kinase
FITC	fluorescein isothiocyanate
GSK3β	glycogen synthase kinase 3β
IL	interleukin
JNK	c-Jun-N-terminal kinase
MAPK	mitogen-activated protein kinase
MMP	matrix metalloproteinase
MC1R	melanocortin 1 receptor
MITF	microphthalmia-associated transcription factor
MSH	melanocyte stimulating hormone
NO	nitric oxide
NF-κB	nuclear factor-κB
Nrf2	nuclear factor erythroid 2-related factor 2
PLGA	poly(D,L-lactide-co-glycolide)
PKA	protein kinase A
PKC	protein kinase C
PKG	protein kinase G
PM	particulate matter
PTEN	phosphatase and tensin homolog deleted on chromosome 10
ROS	reactive oxygen species
SCF	stem cell factor
siRNA	small interfering RNA
SPF	sun protection factor
TNF-α	tumor necrosis factor-α
TPGS	tocopheryl polyethylene glycol 1000 succinate
TUNEL	terminal deoxynucleotidyl transferase dUTP nick end labeling
TYR	tyrosinase
TYRP1	tyrosinase-related protein 1
UV	ultraviolet
UVA-PF	UVA-protection factor
